# Improving Fast EMG Classification for Hand Gesture Recognition: A Comprehensive Analysis of Temporal, Spatial, and Algorithm Configurations for Healthy and Post-Stroke Subjects

**DOI:** 10.3390/s25226980

**Published:** 2025-11-15

**Authors:** Camila Montecinos, Jessica Espinoza, Mónica Zamora Zapata, Viviana Meruane, Ruben Fernandez

**Affiliations:** Department of Mechanical Engineering, Faculty of Physical and Mathematical Sciences, University of Chile, Santiago 850, Chile; jessica.espinoza@ug.uchile.cl (J.E.); mzamora@uchile.cl (M.Z.Z.); vmeruane@uchile.cl (V.M.); rufernan@uchile.cl (R.F.)

**Keywords:** electromyography, classification algorithms, feature extraction, myoelectric control, assistive technology, stroke, dimensionality reduction, machine learning

## Abstract

Electromyography-based assistive and rehabilitation devices have shown potential for restoring mobility, especially for post-stroke patients. However, the variability of biological signals and the processing delays caused by signal acquisition and feature extraction influence myoelectric control systems’ real-time functionality and robustness. This study evaluates the classification performance of electromyographic (EMG) signals for six distinct hand gestures in healthy individuals and post-stroke patients. Different feature extraction methods and machine learning algorithms are employed to analyze the impact of acquisition time (0.5–4 s) and the number of channels (1–4) on model accuracy, robustness, and generalization. The best results are obtained using power spectral density and dimensionality reduction, reaching a classification accuracy of 94.79% with a 2 s signal and 95.31% for 4 s. Acquisition time has a greater effect on accuracy than the number of channels used with accuracy stabilizing at 2 s. We test for generalization using post-stroke patient data, evaluating two scenarios: intra-patient validation with 90% accuracy and cross-patient validation with 35–40% accuracy. This study contributes to developing effective real-time myoelectric control systems for neurorehabilitation.

## 1. Introduction

Stroke is the leading cause of disability and mortality worldwide, often resulting in motor impairments, movement disorders, and muscle spasms that significantly affect an individual’s ability to perform daily activities. Rehabilitation therapy is essential for restoring motor functions and promoting independence. In this context, assistive technologies, such as exoskeletons, are vital in supporting and aiding patients during rehabilitation [1].

The development of adaptive control systems for assistive and rehabilitation devices has increasingly relied on electromyographic (EMG) signals due to their ability to translate muscle activity into actionable commands. EMG-based myoelectric control systems have shown significant potential in controlling motorized devices requiring the coordination of multiple degrees of freedom as in the case of hand articulations [2]. Their application in assistive devices, including prosthetics and orthotics, holds significant potential for enhancing mobility and independence in individuals with motor impairments. A study by Díaz-Grefa et al. [3] highlights that integrating motorized exoskeletons into rehabilitation programs can significantly improve mobility, muscular strength, and overall quality of life. However, there are considerable variations in effectiveness among different users, which underscores the necessity for personalized systems to optimize the performance of these devices.

Over the past decade, pattern recognition methods have become the standard for EMG-based control systems, enabling the classification of muscle activation patterns to facilitate intuitive device control. A good assistive system should be portable and able to classify the movement as fast as possible in order to trigger the assistive motion. Several challenges exist in order to achieve such desired systems. First, the variability of EMG signals, influenced by external and physiological factors, complicates a precise and fast classification, as no universal standard exists for optimizing feature extraction, dimensionality reduction, and classification steps [4]. Second, there is a vast variety of system configurations, especially the number and placement of electrodes, and while having more information enables the use of complex algorithms, it also makes the device less portable. Finally, there is considerable research focused on enhancing the robustness and performance of different signal processing and machine learning algorithms [5]; however, most studies evaluate the classification in a constant time interval, which may limit the direct applicability of their results to real devices.

One key strategy to mitigate the challenges associated with variability in EMG signals is to use dimensionality reduction techniques. These techniques reduce computational costs and enhance class separability, improving classification accuracy and running time. In particular, Principal Component Analysis (PCA) has demonstrated strong potential in optimizing performance. A study by Merzoug et al. [6] evaluated various classification algorithms and demonstrated that implementing PCA as a dimensionality reduction technique can reduce the running time of classification by approximately 10% to 52%, depending on the model, while also improving classification accuracy by 2% to 5%. It is worth noting that other reduction techniques exist that do not solely focus on performance; this is the case of muscle synergy analysis methods, such as Non-Negative Matrix Factorization (NNMF). These methods offer physiologically meaningful insights, especially in stroke-related studies where the interpretability and assessment of motor control are central goals; however, they require a larger number of channels to extract robust and interpretable co-activation patterns, limiting their applicability in small systems.

Before dimensionality reduction, if performed, several options exist to extract signal features and then classify them. Features can be extracted from different domains, including the time (TD), frequency (FD), and time–frequency (TFD), and the choice of domain has a significant impact on classification accuracy. However, due to the non-stationary nature of EMG signals, TD features can be prone to inaccuracies [7]; therefore, FD and TFD features are preferred. In FD, Power Spectral Density (PSD) is particularly advantageous for feature extraction in EMG signals, as it effectively captures key frequency characteristics, helping to identify frequency bands associated with muscle activity. PSD has been commonly used to characterize EMG signals, especially during muscle contractions [8]. On the other hand, Gokgoz and Subasi et al. [9] used Discrete Wavelet Transform (DWT) to decompose sEMG signals into distinct time–frequency components, each corresponding to different frequency bands, and their findings demonstrated that alterations in sEMG signals could be effectively captured through the features extracted from the components of the DWT.

Finally, among commonly used classification algorithms for EMG signals are Linear Discriminant Analysis (LDA), Support Vector Machine (SVM), k-Nearest Neighbor (kNN), and Random Forest (RF), while advanced methods like Artificial Neural Networks (ANNs) are gaining traction [4]. RF has proven effective in classifying hand and wrist movements, leveraging its ensemble learning to enhance robustness against signal variability. Similarly, SVM has demonstrated superior performance over classifiers like kNN in detecting movement patterns, with Dhanjal and Amhia [10] reporting SVM’s higher accuracy and recall, particularly in distinguishing complex movement patterns and generalizing effectively to unseen data. Lee et al. [11] mention that several machine learning methods, including SVM, kNN, and ANN, have achieved over 90% accuracy across multiple studies [12,13,14,15,16,17,18] for 4–10 hand and finger movements.

Despite these achievements, improving accuracy and response time simultaneously remains a challenge in EMG-based systems [11]. Parajuli et al. [19] identify processing delays as a critical limitation, as they compromise the responsiveness required for effective myoelectric control. A review of studies published between 2012 and 2025 [20,21,22,23,24,25,26,27,28,29,30,31] reveals that most classification studies rely on fixed temporal windows of approximately 5 s and use multi-channel EMG configurations ranging from 7 to 24 electrodes with some adopting high-density EMG of up to 128 channels. On the one hand, the achieved accuracy with long fixed intervals may be higher than the actual classification in real time, and this drop could be assessed with progressively shorter time intervals. On the other hand, while these configurations allow for robust signal capture and facilitate comparisons between studies, they also significantly increase computational complexity and latency. This makes them impractical for portable assistive devices that require real-time control.

Building on these considerations, this study considers a hand gesture recognition system with a simple setup of four channels, which is thought to be compatible with a portable device, and it aims to address some of the key limitations in current EMG-based control systems by systematically evaluating how acquisition time and the number of EMG channels affect classification performance. Our goal is to identify configurations that preserve high accuracy while minimizing temporal and spatial requirements, thereby enabling more efficient, responsive, and practical real-time control for assistive and rehabilitation technologies.

To this end, we analyze the impact of two widely used feature extraction methods, PSD and DWT, in combination with dimensionality reduction techniques, PCA and Singular Value Decomposition (SVD), to reduce computational load. Three machine learning classifiers (RF, SVM, and ANN) are assessed across two datasets: one comprising healthy individuals and the other comprising stroke patients, using six functional hand gestures: rest, wrist flexion/extension, grip, finger abduction, and supination. This dual-dataset approach enables the evaluation of the proposed methodology on healthy individuals and stroke patients’ data, allowing us to assess the robustness and generalization capacity of the models using EMG data collected from actual stroke patients. This, in turn, provides insight into their applicability to real-world clinical scenarios.

## 2. Materials and Methods

**Figure 1:**
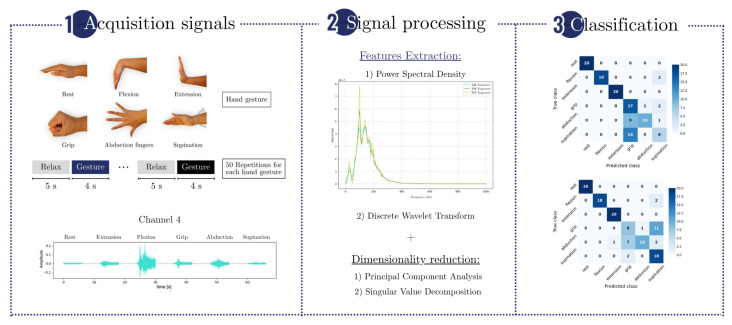
Schematic representation of the implemented methodology. The diagram outlines the key stages of the process, including signal acquisition, signal processing, and classification.

An overview of the methodology is shown in Figure 1, which summarizes the main steps of the experimental pipeline. EMG signals were acquired during the execution of six hand gestures, and features were extracted using PSD and DWT. These features were reduced in dimensionality using PCA or SVD and then classified using RF, SVM, and ANN algorithms. Performance was evaluated under varying acquisition times and channel configurations.

Two datasets were used in this study. The first, described by Ozdemir et al. [30], was employed to assess the impact of different acquisition times (ranging from 0.5 to 4 s) and channel configurations (1 to 4 channels) on classification accuracy. The second dataset, collected from stroke patients, was used to evaluate the generalization of the methodology using the optimal parameters identified in the first stage. In both cases, performance metrics such as accuracy, recall, and precision were calculated.

### 2.1. Dataset

#### 2.1.1. Data from Healthy Individuals

The dataset described by Ozdemir et al. [30] was collected from 40 healthy participants, with an equal distribution of genders, all aged between 18 and 29 years. Information on handedness was also recorded, including 3 left-handed participants, 1 ambidextrous participant, and the remainder being right-handed.

Data acquisition was performed using a BIOPAC MP36 device, capturing information from four channels for each participant’s dominant hand. For classification, six hand gestures were used: rest, wrist extension, wrist flexion, grip, abduction of all fingers, and supination. Data were collected from four distinct surface muscles located near the skin’s surface: extensor carpi ulnaris (channel 1), flexor carpi ulnaris (channel 2), extensor carpi radialis (channel 3) and flexor carpi radialis (channel 4).

EMG data were recorded at a sampling frequency of 2 kHz. The recorded signals had amplitudes ranging from −10 to 10 mV, and they were processed using a bandpass filter (5–500 Hz) and a notch filter (50 Hz) to eliminate noise, including motion artifacts, high-frequency interference, and power line noise.

#### 2.1.2. Data Acquisition of EMG Signals from Stroke Patients

The clinical protocol for EMG signal acquisition in stroke patients was reviewed and approved by the Ethics Committee of the Servicio de Salud Metropolitano Oriente (SSMO), following national legislation (Laws No. 20.120, 19.628, and 20.584) and international ethical standards, including the Declaration of Helsinki (2013), the Belmont Report, the CIOMS guidelines, the Good Clinical Practice (GCP) guidelines, and the Universal Declaration on Bioethics and Human Rights by UNESCO. EMG data were collected at Clínica Los Coihues from two patients diagnosed with chronic ischemic stroke presenting mild to moderate spasticity after obtaining written informed consent from all participants.

**Figure 2:**
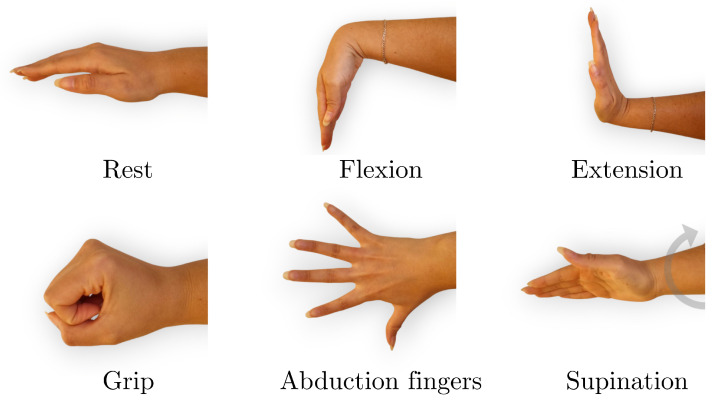
Representation of the six hand gestures used in the EMG data acquisition.

**Figure 3:**
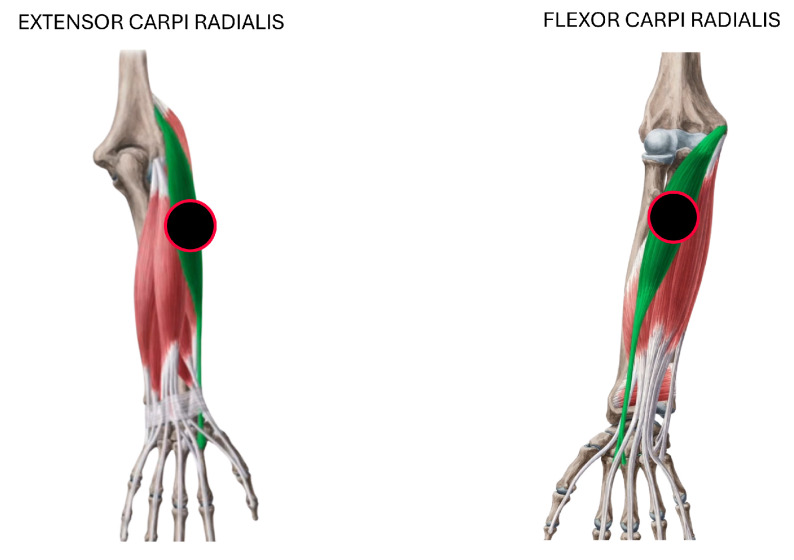
Electrode placement for EMG acquisition. Surface electrodes were positioned following a bipolar configuration over two muscle groups: the flexor carpi radialis and extensor carpi radialis.

The data acquisition process consisted of recording EMG signals during six distinct hand gestures: rest, wrist extension, wrist flexion, grip, abduction of all fingers, and supination (Figure 2). These gestures were selected because they represent a functionally meaningful and diverse set of hand and wrist movements commonly involved in daily activities and rehabilitation routines. They cover a range of motor tasks with varying levels of muscular activation and are particularly relevant for evaluating motor function recovery in individuals with stroke. Surface electrodes were placed on the extensor carpi radialis and flexor carpi radialis muscles to capture muscle activity associated with these movements, as shown in Figure 3.

Data collection followed a structured sequence, as detailed below (see Table 1).

To ensure high-quality EMG signal acquisition, the skin was thoroughly cleaned with alcohol prior to electrode placement to minimize impedance. Disposable surface electrodes (Kendall Meditrace), teardrop-shaped and coated with Ag/AgCl, were used to ensure reliable conductivity and strong adhesion. Additionally, a conductive gel was applied between the skin and electrodes to further optimize electrical contact. A bipolar electrode configuration was implemented to enhance signal clarity and reduce external noise interference. EMG signals were recorded using a two-channel Human SpikerBox device developed by Backyard Brains with a sampling frequency of 2 kHz. The raw signals were processed with a 6th-order Butterworth bandpass filter between 5 and 500 Hz to isolate the relevant EMG components. Additionally, a 50 Hz IIR notch filter was applied to remove powerline interference. Both filters were implemented in Python using the SciPy library and applied in a causal configuration, which was consistent with real-time application constraints. Data acquisition and visualization were carried out using the SpikeRecorder software.

Although the EMG acquisition device does not include a manufacturer-specified measurement error, a technical estimation can be derived from its circuit design. The device employs an INA118 instrumentation amplifier as the initial gain stage, which was followed by RC filtering and additional amplification using operational amplifiers. The overall system gain is approximately 10,000× to 20,000×, allowing microvolt-level EMG signals to be properly amplified for digital acquisition. Based on the amplifier configuration, ADC resolution (10-bit), and typical noise characteristics, the total measurement uncertainty is estimated to fall within a range of 5–20 µV.

### 2.2. Features Extraction

Feature extraction and dimensionality reduction techniques are used to preprocess the data, reducing complexity while retaining the most relevant information. These methods optimize the training of machine learning models and enable the evaluation of their performance.

#### 2.2.1. Power Spectral Density

PSD analysis is a commonly used technique for examining the frequency content of signals. It helps identify dominant frequency components and underlying characteristics. We employ the Welch method for PSD estimation. This approach reduces the variance of the spectral density estimate by averaging successive periodograms. A window size of 256 samples with an overlap of 128 samples is used, offering a balanced trade-off between frequency resolution and variance reduction. The Hanning window is applied to minimize spectral leakage and improve frequency resolution.

#### 2.2.2. Discrete Wavelet Transform

DWT decomposes a signal into sets of coefficients representing its time evolution across specific frequency bands, enabling time and frequency-domain analysis. Among the available wavelet functions, the Daubechies wavelet (db2) demonstrated strong performance for electromyographic signal classification. Various wavelet functions were tested for feature extraction with db2 emerging as one of the most effective in capturing the essential characteristics of EMG signals [32].

The choice of decomposition level in the DWT for EMG signals depends on the sampling frequency and the frequency characteristics of the signal. To determine the optimal decomposition level, the relationship: $f_{N}/2^{n}=f_{d}$ can be applied, where $f_{N}$ is the Nyquist frequency, $f_{d}$ represents the dominant frequency and *n* is the decomposition level.

EMG signals typically contain frequency components between 10 and 500 Hz with a dominant energy concentration in the range of 50 to 150 Hz. Chowdhury et al. [33] analyzed different wavelet functions and concluded that decomposition level 4 provides better performance compared to other levels. This finding aligns with calculations based on the dominant frequency range and the Nyquist frequency, which typically results in an optimal level of 3 to 4 for EMG signals.

#### 2.2.3. Principal Component Analysis

PCA is a dimensionality reduction technique that transforms a dataset into a set of orthogonal components called principal components. These components capture the maximum variance within the data. By simplifying the dataset while preserving its most important features, PCA is beneficial for preprocessing high-dimensional data. In this study, the threshold for the cumulative explained variance was set at 98%, ensuring that the selected principal components retained nearly all the variability in the original dataset.

#### 2.2.4. Singular Vector Descomposition

SVD is a mathematical technique that decomposes a matrix into three components: left singular vectors, singular values, and right singular vectors. This method is used for dimensionality reduction and feature extraction, as it identifies the directions in the data that capture the most variance. Similar to PCA, SVD applies a threshold of 98% cumulative explained variance to retain the most relevant features while discarding less significant information.

### 2.3. Classification Algorithms

Three machine learning models are utilized for EMG signal classification: Random Forest, Support Vector Machine, and Neural Networks. The dataset is split into 20% for testing and 80% for training and validation. A 5-fold cross-validation strategy is employed during training to ensure robust evaluation and reduce the risk of overfitting.

Hyperparameters for each algorithm are optimized using grid search with classification accuracy as the selection metric for identifying the best model configuration. To ensure reproducibility, the random seed (random state) is set to 42 during the grid search process.

#### 2.3.1. Random Forest

RF is an ensemble learning algorithm that builds multiple decision trees using random subsets of data and features to reduce overfitting and variance. For classification, predictions are made by majority vote, and for regression, by averaging the outputs of all trees. To optimize the RF model, a grid search is conducted to tune hyperparameters such as the splitting criterion (e.g., gini or entropy) and the number of trees (n estimators). In a study by Tallapragada and Sagare [34], key hyperparameters, particularly the number of estimators and the splitting criterion, were shown to be critical for improving model performance. Although parameters like maximum tree depth and the number of features considered for each split were also explored, the number of trees and the splitting criterion had the most significant impact on classification accuracy. The hyperparameter ranges used in the grid search for optimizing the RF model include the number of estimators, varying from 1 to 400, and the criterion selection, which can be either Gini or Entropy.

#### 2.3.2. Support Vector Machine

SVM is a supervised learning algorithm that constructs a hyperplane in a high-dimensional space to separate data points of different classes. It is particularly effective for classification tasks, especially in high-dimensional datasets. Key hyperparameters, such as the kernel type and regularization parameter (C), play a critical role in improving SVM performance, as shown in the study by Subasi et al. [35]. Of all the parameters explored, including the polynomial kernel degree and the gamma parameter, the kernel type and C had the most significant impact on classification accuracy. The degree of the polynomial kernel influences the complexity of the decision boundary; in EMG pattern recognition, lower-degree polynomials (e.g., degrees 3 and 5) are often preferred to balance model complexity and generalization, as demonstrated by Kehri and Awale [36]. Table 2 defines the range of hyperparameters used in this study.

#### 2.3.3. Neural Network

NNs are machine learning models inspired by biological neural networks, consisting of interconnected layers of neurons. This study uses a Multi-Layer Perceptron (MLP), which is a type of ANN with an input layer, hidden layers, and an output layer. The MLP is trained using backpropagation to minimize prediction errors.

To further enhance neural network performance, grid search optimization is often employed to tune key hyperparameters, such as the number of hidden layers, the number of neurons per layer, and the learning rate. The ranges for these hyperparameters are determined through prior research and experimentation. Liu and Zhang [37] emphasized that the number of neurons and the learning rate are crucial for improving a model’s generalization ability. While other factors, such as the choice of activation function and regularization parameters, also contribute to performance, the network architecture and learning rate are particularly influential in achieving optimal classification results. The range of hyperparameters used for the regularization parameter alpha spans logarithmically from $10^{-6}$ to $10^{1}$, while the initial learning rate is explored on a logarithmic scale between $10^{-4}$ and $10^{-1}$. Additional hyperparameters and their respective ranges are detailed in Table 3.

### 2.4. Metrics

The performance of the classification models is evaluated using four metrics: accuracy, recall, precision, and F1-score. These metrics give insight into the model’s classification accuracy and handling of imbalanced classes.

- Accuracy: the proportion of correctly classified instances among all instances, calculated globally across all classes.
  $$\mathrm{Accuracy}=\frac{\mathrm{TP}+\mathrm{TN}}{\mathrm{TP}+\mathrm{TN}+\mathrm{FP}+\mathrm{FN}}$$
- Precision: the proportion of true positive predictions among all positive predictions.
  $$\mathrm{Precision}=\frac{\mathrm{TP}}{\mathrm{TP}+\mathrm{FP}}$$
- Recall: the proportion of true positive instances correctly identified.
  $$\mathrm{Recall}=\frac{\mathrm{TP}}{\mathrm{TP}+\mathrm{FN}}$$
- F1-score: The harmonic mean of precision and recall.
  $$F1=2\cdot \frac{\mathrm{Precision}\cdot \mathrm{Recall}}{\mathrm{Precision}+\mathrm{Recall}}$$

Here, the following apply:

TP: true positive;
TN: true negative;
FP: false positive;
FN: false negative.

### 2.5. Case Study Scenarios

Two principal variables are examined to thoroughly assess the classification of EMG signals: temporal window selection and channel configurations. These variables exert a direct influence on the performance of the classification algorithms. We aim to identify the optimal acquisition settings for robust classification performance through a systematic variation of these parameters.

The first variable analyzed was the temporal window duration, which defines the length of the EMG signal segment used for feature extraction and classification. Nine different window lengths were considered: 0.5, 0.6, 0.7, 0.8, 0.9, 1, 2, 3, and 4 s.

The second variable studied was the channel configuration, referring to the number and combination of EMG channels used, which influences the spatial resolution and the information content of the recorded signals. Seven different configurations were analyzed:

- Single-channel configurations: channel 1, channel 2, channel 3, and channel 4.
- Dual-channel configurations: channel 1 and 2, channels 3 and 4.
- Multi-channel configuration: channels 1, 2, 3 and 4.

#### 2.5.1. Global Performance Evaluation

To thoroughly evaluate classification performance, we examines all 63 combinations of temporal windows and channel configurations using a dataset of healthy individuals. Each configuration is tested with five feature extraction methods—PSD, PSD+PCA, PSD+SVD, DWT+PCA, and DWT+SVD—along with three classification algorithms: RF, SVM, and NN. This results in a total of 315 classification experiments. Accuracy is the primary metric for quantifying the overall prediction correctness across the configurations.

#### 2.5.2. Robustness Analysis

(a) Single configuration robustness test: A single temporal window and a specific channel configuration are selected based on the performing combination from the global performance analysis. The dataset is split into 40 train–test partitions for this configuration using previously optimized hyperparameters. The accuracy metric is evaluated across all feature extraction methods (PSD, PSD+PCA, PSD+SVD, DWT+PCA, DWT+SVD) and classification algorithms (RF, SVM, NN).
(b) Multiple configurations robustness test: Three different temporal windows (0.5, 2, and 4 s) and three channel configurations (1, 2, and 4 channels) are evaluated to assess model stability under varying acquisition conditions. For each configuration, only the feature extraction methods that achieved the highest performance in the global evaluation are considered. The three classification algorithms (RF, SVM, NN) are tested with 100 train–test partitions generated per setting. Performance is evaluated based on accuracy, recall, and precision.

#### 2.5.3. Global Performance Evaluation for Stroke Patients Data

For the second dataset, consisting of EMG signals from stroke patients, the temporal parameter is fixed based on the analysis conducted on the dataset of healthy individuals. Specifically, the temporal window that yielded the best results in the global performance evaluation for healthy individuals is selected. Additionally, only one channel of measurement is used for this configuration.

In this scenario, the number of measurements per gesture is varied (20, 30, 40, 50). The feature extraction method that showed the best performance in the previous analysis is applied. Subsequently, the three classification algorithms (RF, SVM, NN) are implemented, and the performance is assessed using the accuracy metric.

#### 2.5.4. Model Generalization Capacity

(a) Intra-patient validation: The temporal and spatial configuration selected from the global performance evaluation on stroke patients’ data is used, but datasets are constructed with 20 and 30 measurements per gesture. The same feature extraction method is applied, and the performance of the three classification algorithms (RF, SVM, NN) is evaluated using accuracy, recall, and precision metrics. This evaluation measures the model’s intra-subject generalization capacity.
(b) Inter-patient validation: The same temporal and spatial configuration, along with the previously selected feature extraction method, is used with datasets containing 50 measurements per gesture. The three classification algorithms are evaluated based on the accuracy metric. This scenario assesses the model’s ability to generalize across different individuals.

### 2.6. Computational Environment

All analyses, including data preprocessing, feature extraction, dimensionality reduction, and classification, were conducted using Python 3.10 within the Spyder IDE. The implementation relied on several open-source libraries, including Numpy (version 2.2.5), Scipy (version 1.15.3), Pandas (version 2.2.3), scikit-learn (version 1.6.1), Matplotlib (version 3.10.3), and PyWavelets (version 1.8.0). Hyperparameter tuning was performed using scikit-learn’s GridSearchCV with cross-validation.

## 3. Results

### 3.1. Sample EMG Readings

**Figure 4:**
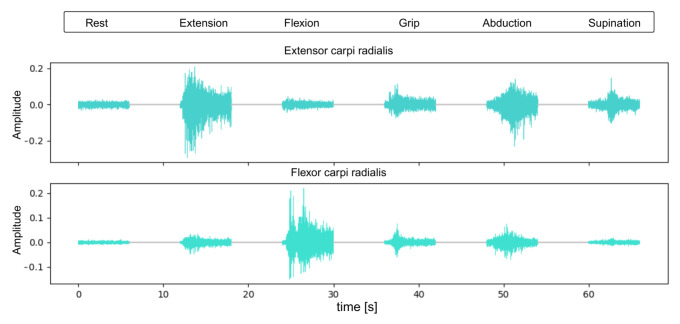
Raw EMG signals recorded from the flexor and extensor carpi radialis muscles during each of the six hand gestures: rest, wrist flexion, wrist extension, grip, finger abduction, and forearm supination.

This section presents representative raw EMG signals for each of the six hand gestures used in the study (Figure 4). The signals illustrate typical patterns and amplitudes recorded from the extensor and flexor muscles.

### 3.2. Global Performance Evaluation

**Figure 5:**
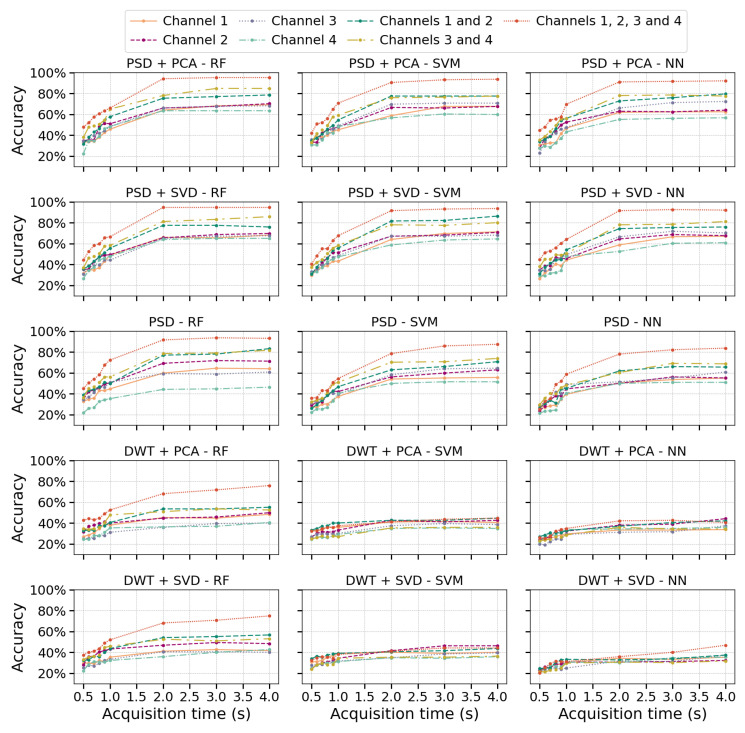
Classification accuracy as a function of acquisition time for different feature extraction methods, algorithms, and measurement channel configurations.

We implemented different feature extraction methods and classification algorithms to evaluate the accuracy of EMG signal classification as a function of acquisition times and combinations of measurement channels. Figure 5 displays the accuracy results obtained from the dataset described by Ozdemir et al. [30], utilizing the 315 configurations outlined in Section 2.5.1. The results shown correspond to the optimal hyperparameters (Appendix A.1) for each model, which were determined through a grid search during the training process.

Figure 5 shows that PSD effectively captures essential frequency-domain information from EMG signals, including energy bands associated with muscle activation. When combined with dimensionality reduction techniques like PCA or SVD, classification accuracy improves consistently across all evaluated algorithms. In contrast, using PSD without dimensionality reduction results in more pronounced differences between algorithms, especially when only one information channel is available, leading to decreased accuracy in SVM and NN. This decline can be attributed to PCA and SVD’s ability to reduce noise and redundancy. Meanwhile, RF demonstrates greater robustness to noise due to its bagging mechanism, which aggregates predictions from multiple trees, reducing variance and improving overall performance.

In the case of DWT+PCA or DWT+SVD, a notable decrease in accuracy is observed compared to PSD-based methods, likely due to the level of decomposition, which can introduce high dimensionality and redundancy in the wavelet coefficients. Interestingly, for shorter temporal windows, DWT performs comparably to other feature extraction methods regardless of the number of channels. However, as the acquisition time increases, its performance does not significantly improve, particularly for SVM and NN, where it remains notably lower than PSD-based methods. In contrast, RF achieves better results with DWT, reinforcing its lower sensitivity to irrelevant signal features. This suggests that the selected wavelet coefficients may contain redundant information, which RF mitigates through its ensemble learning approach, enhancing model robustness.

Increasing the number of channels improved classification accuracy, with a more significant gain observed when increasing from one to two channels compared to the transition from two to four, particularly for PSD, PSD+PCA, and PSD+SVD. Likewise, extending the acquisition time beyond 2 s did not significantly enhance accuracy, as performance tended to stabilize. This plateau may result from the EMG signal stabilizing during sustained contraction, which limits the introduction of new discriminative features. In contrast, the initial phase of muscle contraction exhibits more significant dynamic variations in amplitude and dominant frequencies, which are more informative for class differentiation.

### 3.3. Robustness Analysis

(a) Single configuration robustness test: Table 4 shows the mean accuracy and standard deviation calculated to summarize performance. This metric was evaluated over 40 randomly partitioned datasets with experiments conducted using two channels and a two-second window. All algorithms (RF, SVM, and NN) and feature extraction methods (PSD, PSD+PCA, PSD+SVD, DWT+PCA, and DWT+SVD) were tested.

The coefficient of variation (CV) is used to evaluate the robustness of the models, comparing the standard deviation to the mean. Most methods show a low CV, typically below 5%, with PSD methods demonstrating consistent performance. However, methods like DWT+SVD and DWT+PCA exhibit higher variability with CVs between 5 and 10% across all algorithms. Notably, the NN algorithm has the highest CV across all feature extraction methods, indicating greater sensitivity to data variability. Dimensionality reduction techniques like PCA improve robustness, as seen in the lower CVs for PSD+PCA (2.9–3.5%), while DWT methods show higher CVs, especially DWT+SVD (6.1–10.5%), indicating more variability in performance.

Mean and standard deviation were used to summarize model performance across repetitions. To confirm the validity of these metrics, a normality analysis was performed using the Shapiro–Wilk test, which indicated that the classification results approximately follow a normal distribution (*p*-value > 0.05). While a few configurations yielded *p*-values slightly below this threshold, the objective of this analysis is descriptive rather than inferential, as no statistical hypothesis testing is conducted at this stage. To complement the statistical test, Q–Q plots were also examined, showing that the empirical distributions exhibit a near linear alignment with the expected normal quantiles, supporting the assumption of approximate normality.

Therefore, the use of mean and standard deviation remains appropriate for assessing model robustness through the coefficient of variation (CV), which provides a standardized measure of variability relative to the mean. To further examine the stability of model performance under varying temporal and spatial configurations, boxplots were also used to report the full distribution of classification results across multiple data splits, offering a complementary visual assessment of robustness.

**Figure 6:**
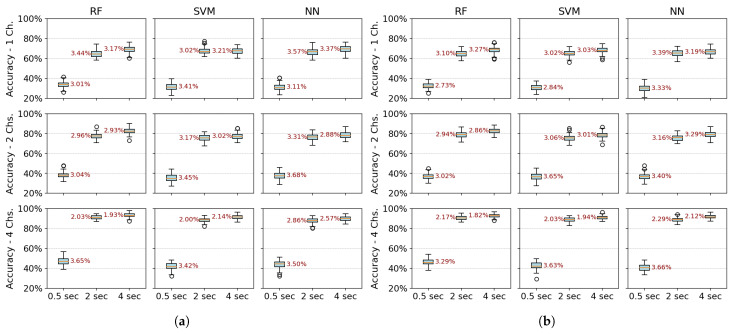
Boxplots of average classification accuracy over 100 iterations, illustrating the impact of acquisition times (0.5 s, 2 s, and 4 s) on the performance of RF, SVM, and NN algorithms across different numbers of channels. Standard deviation values are displayed next to each box. (**a**) PSD-PCA feature extraction methods (**b**) PSD-SVD feature extraction methods.

**Figure 7:**
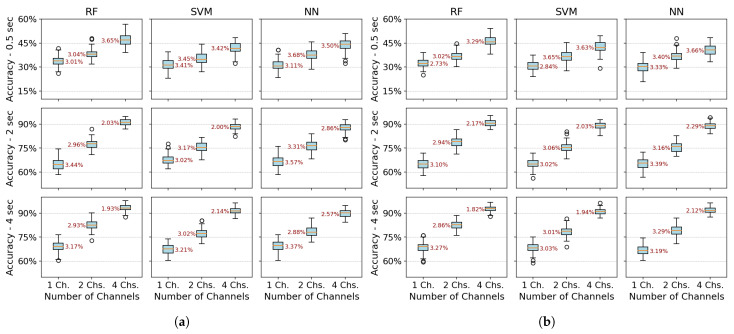
Boxplots of average classification accuracy over 100 iterations, illustrating the impact of the number of channels (1, 2, and 4) on the performance of RF, SVM, and NN algorithms across different acquisition window lengths (0.5, 2, and 4 s). Standard deviation values are displayed next to each box. (**a**) PSD-PCA feature extraction methods. (**b**) PSD-SVD feature extraction methods.

(b) Multiple configuration robustness test: Figure 6 and Figure 7 present boxplots of accuracy for three spatial configurations (single channel, 2 channels, and 4 channels) and three temporal window configurations (0.5, 2, and 4 s of acquisition time). For the single-channel setup, channel 3 (extensor carpi radialis) is used, as it demonstrates the best individual performance. In the two-channel configuration, channels 3 and 4 (extensor and flexor carpi radialis muscles, respectively) are employed. The four-channel setup combines channels 3 and 4 (extensor and flexor carpi radialis) with channels 1 and 2 (extensor and flexor carpi ulnaris muscles). The robustness of the models was evaluated using 100 randomly partitioned datasets. The analysis focuses on feature extraction methods that showed the best performance, namely PSD+PCA and PSD+SVD—across all algorithms (RF, SVM, and NN).

Figure 6 more clearly demonstrates that accuracy does not improve beyond 2 s of acquisition time. Furthermore, the data dispersion—reflected in narrow interquartile ranges—and the standard deviation remain stable after this point, showing no significant variations. With four channels, all algorithms show robust performance (low variability–CV: 2–3%). RF marginally outperforms SVM/NN in two-channel configurations. Single-channel data further amplify variability, underscoring the impact of spatiotemporal limitations on model stability.

Figure 7 reveals a critical interplay between temporal and spatial information in EMG classification. For short acquisition windows (0.5 s), increasing the number of channels from 1 to 4 yields only marginal accuracy gains (∼10%), suggesting that the additional data provided by more channels are insufficient to compensate for the lack of relevant temporal dynamics in the signal. Conversely, with longer windows (2–4 s), channel count becomes decisive, boosting accuracy by up to 22% as more dynamic signal features are captured. Notably, shorter windows also exhibit higher data dispersion (wider IQRs), while longer acquisitions coupled with more channels reduce variability, reflecting improved robustness.

The comparative analysis reveals temporal acquisition as the dominant factor (Figure 6 and Figure 7). A single channel achieves ∼30% accuracy gain when the temporal window increases from 0.5 s to 2 s, surpassing any multi-channel advantage at shorter intervals. This underscores that dynamic temporal features are fundamental for discrimination, while channels primarily refine results once sufficient temporal information is attained.

**Figure 8:**
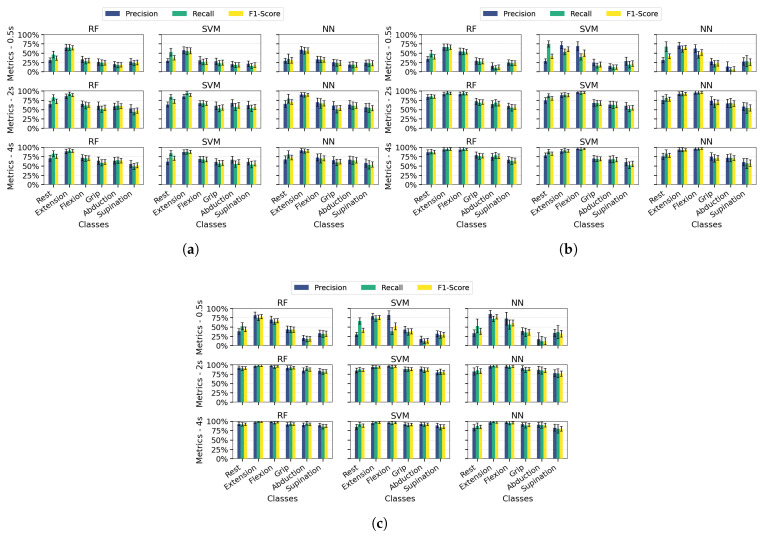
Average precision, recall, and F1-score for PSD-PCA feature extraction across different numbers of measurement channels, evaluated over 100 iterations for RF, SVM, and NN algorithms at different acquisition times (0.5 s, 2 s, and 4 s). Error bars represent the standard deviation of the metrics across iterations. (**a**) One-channel metrics. (**b**) Two-channel metrics. (**c**) Four-channel metrics.

(c) Classification performance by type of motion:

Figure 8 presents each class’s average precision, recall, and F1-score values in bar plot format with error bars indicating the standard deviation across 100 dataset splits. Results are shown for the PSD-PCA method (with PSD-SVD exhibiting similar patterns) under the previously defined temporal windows and channel configurations.

Extending the analysis to class-specific performance, wrist extension consistently outperformed other classes across all channel configurations (highest precision/recall/F1), which was attributable to the distinctive EMG signatures from extensor carpi radialis (channel 3). While multi-channel configurations improved the overall metric consistency, three limitations became apparent: (1) supination exhibited marginally lower performance than other classes even with optimal conditions (≥2 s windows and multi-channel spatial resolution), (2) finger abduction demonstrated degraded classification in short windows (0.5 s)—which was likely from suboptimal electrode positioning relative to intrinsic hand muscles, and (3) the rest class showed biased performance in SVM/NN (high recall but low precision) due to systematic confusion between inactive states and low-intensity movements. These findings indicate that while proximal muscle movements benefit from channel redundancy, reliable distal movement classification requires both anatomical electrode optimization and appropriate temporal windows.

Across most configurations, standard deviations remained relatively stable, indicating consistent performance throughout the evaluation iterations. However, higher deviations occurred in scenarios with unbalanced metrics, mainly when temporal information was limited. Supination demonstrated slightly more significant standard deviations than other classes, suggesting greater variability in movement execution patterns. Among the algorithms, NN showed the least robustness with higher deviation values associated with individual class performance metrics.

### 3.4. Global Performance Evaluation for Stroke Patients Data

**Figure 9:**
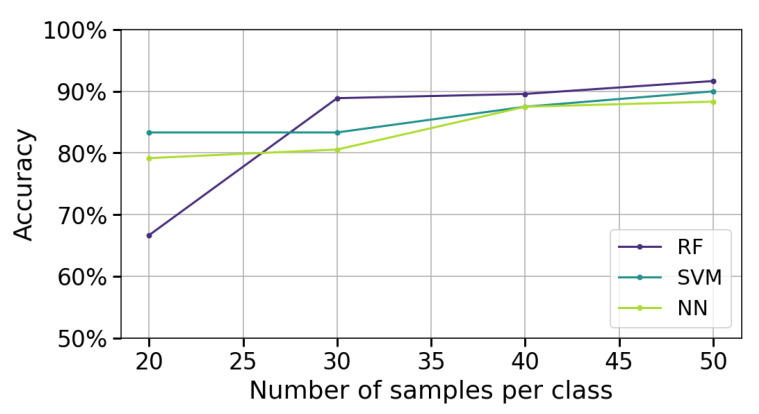
Accuracy as a function of the number of samples per class for RF, SVM, and NN algorithms using PSD-PCA feature extraction with a single measurement channel (channel 4) and a temporal window of 2 s.

Figure 9 presents the classification performance of the algorithms as the number of measurements per class varies (20, 30, 40, and 50). The analysis uses stroke patient data to evaluate performance in a realistic application scenario. PSD-PCA is employed for feature extraction with a 2 s acquisition window, which were both chosen based on previous results that demonstrated the best performance. A single measurement channel (channel 4) is used.

In Figure 9, it can be observed that as the number of measurements per class increases (from 20 to 50), there is a consistent improvement in the accuracy of all three algorithms. This behavior indicates that a larger amount of data per class provides more representative information and reduces prediction variability. Between 40 and 50 measurements per class, the rate of improvement in accuracy diminishes, suggesting the models are approaching their asymptotic performance limits with the given feature space and architecture.

At 20 measurements per class, RF exhibits a pronounced accuracy decline compared to SVM and NN. This behavior aligns with RF’s ensemble nature; its majority-voting mechanism becomes unreliable when constituent trees are trained on insufficient, non-representative subsets. In contrast, SVM and NN maintain relatively stable performance through their inherent regularization mechanisms, which better accommodate limited training data by explicitly optimizing the bias–variance tradeoff.

### 3.5. Model Generalization Capacity

Table 5 and Table 6 present intra-patient validation results, comparing model training performance with validation on 20 unseen measurements from the same subject. Using the same spatiotemporal configuration and feature extraction methods, we evaluate generalization at 20/30 measurements per class through metric accuracy across all algorithms.

Table 5 and Table 6 reveal three key findings about algorithmic performance with increasing training data (20 to 30 measurements per class). First, all models show improved validation accuracy, as expected, with RF demonstrating the largest absolute gains (though its significant training–validation gap suggests overfitting tendencies). Second, NN achieves perfect training–validation alignment at 30 measurements, indicating exceptional generalization when sufficient data capture class variability. Third, SVM maintains the most stable performance across both data regimes, with modest but consistent improvements and the smallest generalization gaps, reflecting its inherent regularization strengths.

**Figure 10:**
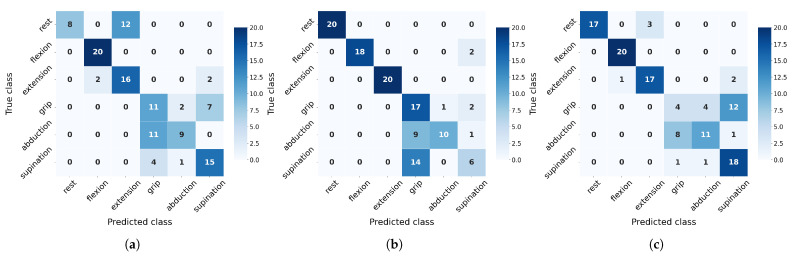
Confusion matrix for validation with 20 measurements per class evaluated across algorithms: (**a**) RF, (**b**) SVM, and (**c**) NN.

**Figure 11:**
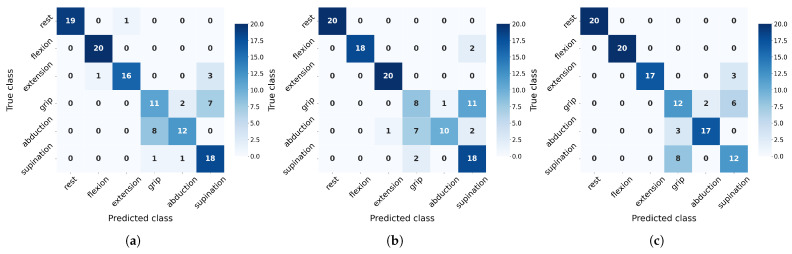
Confusion matrix for validation with 30 measurements per class evaluated across algorithms: (**a**) RF, (**b**) SVM, and (**c**) NN.

On the other hand, to evaluate class-wise generalization capacity, we analyze precision and recall metrics derived from confusion matrices (Figure 10 and Figure 11). These matrices reveal distinct misclassification patterns across movement categories, providing crucial insights into which specific gesture confusions most impact overall performance and how sensitivity to particular movements varies between algorithms under different training conditions.

The confusion matrices (Figure 10 and Figure 11) reveal distinct class-specific patterns across algorithms. Rest, flexion, and extension consistently achieve high F1-scores due to their distinctive EMG signatures, particularly benefiting from flexor carpi radialis activation (channel 4). In contrast, grip, abduction, and supination present greater challenges with their performance varying significantly by algorithms: SVM overpredicts grip (85% recall but 42% precision at 20 measurements) while struggling with supination (F1 = 39%), though the latter improves substantially with more data (F1 = 68%). NN shows opposite tendencies, severely underdetecting grip (20% recall) but overpredicting supination (90% recall, 55% precision). RF exhibits unique rest-class behavior (100% precision but 40% recall), suggesting conservative prediction patterns. Crucially, all algorithms show measurable improvement in problematic classes when increasing from 20 to 30 measurements, confirming that additional training data helps resolve ambiguous EMG patterns, though fundamental feature limitations remain for certain movements.

### 3.6. Evaluation of Cross-Patient Generalization

When the models were validated using data from other patients, all three algorithms showed comparable accuracy scores, ranging from approximately 35% to 40%. This represents a significant decrease compared to validation with data from the same participants. One possible explanation is that the EMG signal characteristics vary substantially across individuals (e.g., due to differences in muscle anatomy or electrode placement), reducing the models’ generalization ability.

This underscores the crucial role of collecting more diverse datasets or applying transfer learning and domain adaptation strategies to mitigate inter-patient variability. However, the need for such approaches can depend heavily on the specific application. For instance, in the context of myoelectric control for assistive or rehabilitation devices, it may be sufficient—and even advantageous—to train and fine-tune the model using the target user’s signals. By doing so, the system can adapt more effectively to the unique EMG patterns of that individual, thereby enhancing generalization and performance under real conditions.

## 4. Discussion

This study provides a systematic assessment of how acquisition configuration, specifically signal duration and the number of EMG channels, influences classification performance. The results demonstrate that with just two channels and a 2-s acquisition window, it is possible to achieve accuracies around 95%, which is consistent with prior studies that report over 90% accuracy using traditional classifiers such as RF, SVM, and ANN on EMG datasets with comparable gestures [12,13,14,15,16,17,18]. These findings confirm that high-density EMG setups are not strictly necessary to achieve strong classification performance, aligning with the goal of developing efficient and portable myoelectric control systems.

A comparative analysis of feature extraction methods showed that PSD consistently outperformed DWT in terms of classification accuracy across all classifiers. This observation aligns with previous work indicating that PSD effectively captures the frequency characteristics of muscle activity, especially during contraction phases [8]. The inferior performance of DWT, particularly for SVM and ANN, may be due to sensitivity to irrelevant wavelet coefficients when decomposition levels are not optimally tuned. In contrast, RF showed greater robustness when used with DWT, which was likely due to its ensemble structure and inherent feature selection.

Dimensionality reduction techniques, particularly PCA and SVD, contributed to notable improvements in classification accuracy and computational efficiency. These findings support the results of Merzoug et al. [6], who reported that PCA improved classification accuracy by 2–5% while reducing processing time by up to 52%. In our study, performance improvements from dimensionality reduction varied depending on data richness: for limited configurations (e.g., 1–2 channels, 0.5 s windows), the gain ranged from 2% to 10%, whereas richer configurations (e.g., 4 channels or 2 s windows) achieved up to 15% improvement in accuracy. These gains underscore the relevance of dimensionality reduction for practical, low-latency systems.

Regarding the number of channels, we observed diminishing returns beyond two electrodes. Extending the acquisition window from 0.5 to 2 s yielded substantial accuracy gains, with minimal improvement beyond that, suggesting that the most informative EMG content lies in the early activation phase.

When applied to stroke patient data, the optimal configuration identified in healthy subjects (2 s window, PSD+PCA, 1–2 channels) achieved strong intra-patient classification results, reaching 80–90% accuracy depending on the training set size. In contrast, cross-patient validation yielded lower accuracy levels, around 35–40%, highlighting the impact of inter-subject variability. These results suggest the feasibility of implementing patient-specific myoelectric control in assistive devices using EMG signals from the affected limb. Training models individually for each user helps mitigate the variability associated with differences in motor impairment and spasticity levels, which is a key limitation for general-purpose systems. As an alternative strategy, biomimetic control could be employed using data from the unaffected limb, leveraging greater data availability and symmetry to enhance usability and reduce calibration time in practical applications.

## 5. Conclusions

This work demonstrates that accurate EMG-based classification can be achieved using reduced temporal and spatial configurations, making it viable for real-time applications. Feature extraction using PSD, combined with dimensionality reduction (PCA or SVD), provides consistent performance across classifiers while significantly reducing computational load. The findings validate that simplified configurations—such as two channels and 2 s windows—are sufficient to achieve classification accuracies comparable to more complex setups, especially in healthy subjects and intra-patient stroke scenarios. This supports the development of practical and adaptive myoelectric interfaces for rehabilitation and assistive technologies.

Future research should explore the integration of deep learning architectures capable of learning robust representations from raw or minimally processed EMG data, potentially enhancing classification accuracy and adaptability. Real-time validation, particularly with stroke patients, is critical to evaluate the system’s responsiveness and stability under dynamic, non-controlled conditions. Additionally, incorporating user-centered metrics, such as comfort, perceived responsiveness, and ease of calibration, will be essential to transition from experimental prototypes to clinically viable assistive technologies tailored to users’ functional needs.

## Figures and Tables

**Table 1:** EMG data acquisition protocol detailing the sequence of data collection.

Phase	Duration	Description
Preparation	∼3 min	Electrode placement, signal calibration, and subject familiarization.
Gesture task	4 s per trial	The subject maintains the specified gesture while EMG data are recorded.
Rest period	5 s	The subject relaxes before the next trial to prevent fatigue.
Repetitions	50 per gesture	Each gesture is repeated 50 times for consistency.
Total session time	∼1 h per patient	Including preparation, trials, and rest periods.

**Table 2:** Hyperparameter ranges used in the grid search for SVM model optimization.

Parameter	Range
Kernel	{rbf, poly}
Gamma	logspace (−5, 0, 50)
C	logspace (−1, 4, 80)
Degree	{3, 4, 5}

**Table 3:** Hyperparameter and setup used in the grid search for NN model optimization.

Hidden layers	(64), (128), (256), (64, 64), (128, 128), (256, 128), (128, 64), (128, 64, 32)
Function activation	{tanh, relu, logistic}
Solver	{adam, sgd}

**Table 4:** Mean accuracy and standard deviation for 40 dataset splits, using two channels and a two-second window length, for different feature extraction methods and all algorithms.

		PSD	PSD + PCA	PSD + SVD	WT + PCA	WT + SVD
Mean accuracy (±Std)	RF	(78.5 ± 2.9)%	(78.4 ± 2.7)%	(80.1 ± 2.9)%	(47.7 ± 2.3)%	(49.0 ± 3.0)%
	SVM	(64.1 ± 3.4)%	(75.8 ± 2.9)%	(78.8 ± 3.1)%	(43.4 ± 2.5)%	(38.1 ± 3.3)%
	NN	(61.1 ± 3.7)%	(76.2 ± 3.1)%	(77.7 ± 3.1)%	(32.3 ± 2.9)%	(31.4 ± 3.3)%

**Table 5:** Training and validation accuracy for 20 measurements per class across RF, SVM, and NN algorithms.

	Accuracy Training	Accuracy Validation
RF	67%	66%
SVM	83%	76%
NN	79%	73%

**Table 6:** Training and validation accuracy for 30 measurements per class across RF, SVM, and NN algorithms.

	Accuracy Training	Accuracy Validation
RF	89%	80%
SVM	83%	78%
NN	81%	81%

## Data Availability

The data for stroke patients are not publicly available due to ethical and privacy restrictions involving patient information. However, the dataset for healthy individuals that was used in this study is publicly available and can be accessed from the source [30].

## Abbreviations

The following abbreviations are used in this manuscript:

EMG	electromyography
TD	time domain
FD	frequency domain
TFD	time–frequency domain
DWT	Discrete Wavelet Transform
PCA	Principal Component Analysis
PSD	Power Spectral Density
SVD	Singular Value Decomposition
RF	Random Forest
SVM	Support Vector Machine
NN	Neural Network
LDA	Linear Discriminant Analysis
kNN	k-Nearest Neighbors
ANN	Artificial Neural Network
MLP	Multi-Layer Perceptron

## Appendix A. Hyperparameters

### Appendix A.1. Hyperparameters Healthy Individuals

The results of the best hyperparameters identified through grid search for the evaluated models are presented. This analysis examines whether clear trends can be observed across various feature extraction methods and model types with an emphasis on the configurations that consistently yield optimal performance.

- The RF model, across all feature extraction methods, demonstrates commonalities and distinctions in hyperparameter behavior. One significant observation is the absence of consistent trends in the selection of the partitioning criterion. Both ‘gini’ and ‘entropy’ are chosen interchangeably, with no evident preference, suggesting that the optimal criterion is largely influenced by the specific dataset and configuration rather than the feature extraction method employed.
  Regarding the number of estimators, no strong correlation was found with the window duration for any method. However, a recurring trend emerges when examining the influence of channel combinations. Configurations utilizing multiple channels often required a larger number of estimators, which is likely due to the increased variability and complexity of the feature space introduced by combining signals from different channels.
  In the vast majority of cases, regardless of the feature extraction method, the number of estimators typically ranged between 100 and 250. This medium-to-high range of trees indicates that RF models generally benefit from larger forests, which can better capture the variability and complexity of the EMG signals across different configurations.
- SVM models exhibit distinct trends in hyperparameter selection across the various feature extraction methods. For the kernel, rbf predominates across all methods with occasional use of the poly kernel in PSD–PCA and PSD–SVD. When poly is selected, the degree is typically set to 3 or 5.
  For the regularization parameter C, values vary depending on the feature extraction method. In PSD–PCA and PSD–SVD, C ranges from approximately 100 to 8000, reflecting lower regularization and a stronger penalization of classification errors. WT–PCA and WT–SVD favor smaller values, typically between 1 and 400, indicating higher regularization and a greater tolerance for misclassifications to maintain simpler decision boundaries. PSD without dimensionality reduction shows a preference for values in the range of 1 to 100, suggesting that raw PSD features generally require higher regularization with lower penalization of errors.
  For the gamma parameter, PSD–PCA and PSD–SVD typically use values between $10^{-3}$ and $10^{-1}$. Higher gamma values are observed for channel 4, which is likely due to its signals exhibiting lower variability or less distinguishable patterns. These higher values create tighter kernel focus, enabling more detailed decision boundaries but increasing the risk of overfitting. A notable trend is that as the number of channels increases, gamma tends to decrease. The additional information from multiple channels provides redundancy and complementarity, allowing the model to generalize better with broader, less localized decision boundaries. In WT–PCA and WT–SVD, gamma values are considerably smaller, often concentrated around $10^{-3}$. For PSD without dimensionality reduction, most gamma values cluster between $10^{-3}$ and $10^{-2}$.
- ANN models exhibit consistent trends in hyperparameter selection across the various feature extraction methods with notable differences influenced by the data complexity and feature extraction approach. For the regularization parameter alpha, values are concentrated in similar ranges for most methods, typically between $10^{-4}$ and $10^{-2}$. These ranges reflect varying levels of constraint to balance model complexity and overfitting with PSD methods favoring more constraint compared to wavelet-transformed features.
  The network architecture, configurations with two or three hidden layers dominate across all methods, with frequent patterns such as (128, 64, 32), (128, 64), and (128, 128). Networks with a single large layer, such as (256), are also observed but less frequently, often appearing in cases with simpler data structures or fewer channels. Increasing the number of channels or extending the window duration generally coincides with deeper or more neuron-dense architectures, though simpler configurations (e.g., (128, 64)) persist in some multi-channel setups.
  The activation function relu is consistently predominant across all methods due to its efficiency and robustness in classification tasks. However, logistic and tanh occasionally appear, particularly in PSD-only methods and configurations with shorter windows or fewer channels. These alternatives favor in specific cases where the variability of the signal requires smoother or more gradual activation.
  The learning rate is generally concentrated in the range of $10^{-3}$ to $10^{-2}$ for all feature extraction methods, striking a balance between convergence speed and stability during training. Solver selection is largely uniform with adam dominating across all configurations due to its adaptive nature and effectiveness in optimizing complex networks. The occasional use of sgd is observed in specific setups.

### Appendix A.2. Hyperparameters Stroke Patients

- For RF models, the optimal number of estimators varies between 98 and 197 across the training datasets with a trend of using larger forests as the number of measurements decreases. This suggests that with less data, the model compensates by relying on more trees to achieve stable decision boundaries. The criterion alternates between gini and entropy with no clear preference.
- For SVM models, the kernel rbf consistently dominates across all configurations, highlighting its ability to handle the non-linear relationships inherent in EMG data. The regularization parameter C varies from approximately 20 to 61, with a slight tendency to increase as the number of measurements decreases, indicating reduced regularization to better capture the variability in smaller datasets. The gamma parameter ranges from 0.001 to 0.008, with smaller values typically appearing in datasets with fewer measurements, allowing for broader decision boundaries in the higher-dimensional feature space.
- For ANN models, the activation function relu is consistently selected with alpha values concentrated between $10^{-4}$ and $10^{-2}$. For the architecture, networks with two or three layers dominate with configurations such as (128, 128), (128, 64), and (128, 64, 32). The networks tend to become slightly deeper or denser as the number of measurements increases, suggesting that larger datasets allow for more complex models. The learning rate falls within the range of 0.02 to 0.05, balancing convergence speed and stability.
